# Cognitive performance after ischaemic stroke

**DOI:** 10.1590/1980-57642015DN92000011

**Published:** 2015

**Authors:** Maria Gabriela R. Ferreira, Carla Heloísa C. Moro, Selma C. Franco

**Affiliations:** 1 Psychologist, Specialist in Neuropsychology, Masters in Health and the Environment from the Regional University of Joinville, Professor at the Departments of Psychology and Medicine of the Regional University of Joinville (UNIVILLE), Joinville SC, Brazil.; 2 Neurologist, MD, Professor at the Department of Medicine of the Regional University of Joinville (UNIVILLE), Joinville SC, Brazil.; 3 Public Health Specialist, MD, Professor at the Department of Medicine of the Regional University of Joinville (Univille), Joinville SC, Brazil. PhD in Child and Adolescent Health from Unicamp and post-doctorate from Rovira i Virgili University, Tarragona, Spain.

**Keywords:** stroke, cognition, depression, disabled persons, neuropsychology

## Abstract

Cognitive impairment after stroke affects the patient recovery process. Therefore, the identification of factors associated with cognitive outcomes is important since it allows risk profiles of stroke survivors to be determined.

**Objective:**

To assess cognitive outcome of stroke outpatients and investigate associations among clinical and demographic variables, vascular risk factors, depression symptoms and functional ability; and to describe the neuropsychological profile of these patients.

**Methods:**

A cross-sectional design study was conducted. Subjects who suffered a first-ever ischaemic stroke 6 to 10 months prior to data collection underwent neuropsychological assessment and screening for depressive symptoms and functional ability. The outcome "cognitive performance" was analyzed considering two groups: "cognitive impairment" and "no cognitive impairment".

**Results:**

There was a statistically significant association between cognitive impairment and female gender, age, stroke severity and functional ability. Regarding neuropsychological profile, the cognitive impairment group exhibited more generalized deficits in attention, visuospatial organization, verbal functions and verbal memory domains compared to the community control group.

**Conclusion:**

The occurrence of cognitive impairment among patients was high, especially in women, older participants, individuals with more severe stroke, and greater impairment in functional ability. Multiple cognitive domains are affected and this may hamper recovery and negatively impact independence and quality of life after stroke.

## Abstract (pt)

O prejuízo na cognição após evento de Acidente Vascular Cerebral Isquêmico (AVCI) afeta a recuperação dos pacientes. Dessa forma, a identificação de fatores associados ao desfecho cognitivo torna-se relevante ao permitir traçar perfis de risco para os pacientes acometidos.

**Objetivo:**

Avaliar o desfecho cognitivo dos pacientes vitimados por AVCI em seguimento ambulatorial, verificar associação com variáveis clínicas e demográficas dos pacientes, sintomas de depressão e capacidade funcional, e descrever seu perfil neuropsicológico.

**Métodos:**

Foi realizado um estudo transversal, cuja amostra foi composta por 45 pacientes que apresentaram primeiro evento de AVCI nos 6 a 10 meses anteriores à coleta de dados. Os sujeitos foram entrevistados e submetidos à avaliação neuropsicológica e da capacidade funcional e à verificação da presença de sintomas de depressão. O desfecho "desempenho cognitivo" foi analisado considerando dois grupos: "com prejuízo cognitivo" e "sem prejuízo cognitivo".

**Resultados:**

Identificou-se associação estatisticamente significativa entre prejuízo cognitivo e sexo feminino, idade, gravidade do AVC e capacidade funcional. Considerando o perfil neuropsicológico, o grupo de sujeitos que apresentou prejuízo cognitivo exibiu déficits mais generalizados, nos domínios de atenção, organização visuoespacial, funções verbais e memória verbal, quando seu desempenho foi comparado aos controles da comunidade.

**Conclusão:**

A ocorrência de prejuízo cognitivo entre os pacientes é elevada, especialmente em mulheres, pessoas com maior idade, naqueles que sofreram AVC mais graves e naqueles que mostraram maior prejuízo na capacidade funcional. Diversos domínios cognitivos são afetados. Isto pode dificultar a recuperação, a independência e a qualidade de vida após o AVC.

## INTRODUCTION

The cognitive sequelae caused by stroke can lead to serious deficits, such as disability, impairment and reduced quality of life.^1^ The brain is able to reorganize, resulting in cognitive improvement in the first few months after stroke.^2^ However, some patients can evolve without improvement or even deteriorate cognitively in the long term,^2-4^ going on to develop vascular dementia.

Identifying the factors associated with cognitive outcome and the occurrence of dementia is important in the follow-up of stroke survivors in as far as this allows a risk profile to be established for each patient. In this line of investigation, Del Ser et al. (2005) identified age, previous cognitive decline, polypharmacy and hypotension on admission as risk factors for progression of cognitive impairment in patients suffering stroke.^5^ Nys et al. (2005) found diabetes mellitus to be associated as a factor for worse cognitive recovery six months after stroke, representing a risk factor for vascular dementia.^2^

The possible clinical outcomes post-stroke include: death, survival, impairment, disablement and deficits.^6^ Against this background, long-term and large-scale population-based follow-up studies assessing the neuropsychological outcomes and their prognostic value are needed,^6^ as well as functional outcomes, exploring the intricate relationship between physical functioning (impairment), activity (disability) and participation (deficit and quality of life). Based on such studies, long-term outcomes can be estimated allowing valuable information to be provided to stroke survivors and their family members, and also to healthcare systems for treatment planning. The identification of predictive factors can contribute toward developing preventive strategies and rehabilitation.^7^

Almost half of stroke survivors present neuropsychological deficits, but few studies have investigated neuropsychological sequelae as a stroke outcome.^7^ Barker-Collo et al. (2006), in a review article on the impact of neuropsychological deficits in the functional outcome of stroke, found scant adequate data available on the neuropsychological profile associated with different stroke subtypes.^6^ Moreover, some of the studies available have major methodological limitations, failing to perform comprehensive neuropsychological assessment or exhibiting selection bias.^7^

The magnitude of stroke, an event affecting a large number of people worldwide every year, makes it important for patients and their families to be aware of their future possibilities. This information encompasses not only the potential benefits to the patient of rehabilitation in physical, cognitive and psychological terms, but also the need to reorganize their lives (personal, social and occupational). Knowledge yielded by studies on motor, cognitive and functional outcome can help teams of professionals who deal with stroke to guide patients and their family members.^6^

In this context, the objectives of this study were to assess the cognitive outcome of first-ever stroke patients undergoing outpatient treatment; to ascertain the association of outcome with clinical and demographic variables, depression symptoms and functional ability; and to describe their neuropsychological profile.

## METHODS

**Casuistic.** The present cross-sectional design study was performed in 45 patients followed at a Neurology Outpatient Clinic specialized in managing stroke patients after discharge from a public hospital with a Stroke Unit.

**Ethics.** All patients agreeing to take part in the study signed the Informed Consent Form. None of the patients meeting the inclusion criteria refused to participate in the study. The study project was approved by the Research Ethics committee of the Regional University of Joinville under process no. 110/09.

**Subject selection.** A total of 45 patients undergoing outpatient treatment, all of whom had suffered first-ever single strokes 6-10 months prior, were studied between July 2009 and 2010.

At the service of origin, a local referral hospital for treating Stroke, patients arriving at the emergency room presenting stroke are admitted to the Stroke Unit (U-AVC) which has 21 beds, without the application of any exclusion criteria such as age, severity or type of stroke. The criteria for stroke diagnosis are based on the presence of acute focal deficit, confirmed by Computed Tomography disclosing lesion associated with the deficit presented (NINDS criteria).^8^ The classification of Stroke subtype is performed by a neurologist after correlating clinical data (BAMFORD classification),^9^ the usual laboratory routine for investigating cerebrovascular disease, topographic assessment of imaging exams (cranial computed tomography and/or MRI) and functional exams (echo Doppler of the carotids and vertebral arteries, transcranial Doppler, and echocardiogram). The investigation of possible subcortical involvement is performed by imaging analysis. The physiopathologic diagnosis of stroke subtype was protocoled according to the TOAST study.^10,11^ All stroke patients have access to thrombolysis with recombinant tissue plasminogen activator (rt-PA), via intravenous and endovenous routes. In the city, the rate of thrombolysis incidence in 2010 was 9.4 per 100,000 inhabitants.^12^ Having excluded atherothrombotic cases, investigation of Atrial Fibrillation, including paroxysmal, takes place using electrocardiogram (repeated when required) and cardiac holter for at least 24 hours.

Entry to the hospital takes place normally via the Emergency Ambulance Service (SAMU) which has treated patients since 2005.^12^ The population of the city is a target of educational and prevention campaigns for stroke which allows symptoms to be recognized quickly and assistance sought. Patients discharged from the U-AVC of the service are selected for specific outpatient follow-up, involving a neurologist specialized in neurovascular medicine, based on the following criteria: preference given to younger patients, with rare stroke etiologies (thrombophilia, non-atherosclerotic vasculitis); individuals with difficulties accessing other outpatient follow-up services in the community, thereby ensuring adherence to treatment and access to rehabilitation in the event of further disablement following discharge; when patient is submitted to endarterectomy of the internal carotid or thrombolysis with rt-PA. Remaining patients discharged from the U-AVC of the service are referred to general neurology outpatient units in the city.

The inclusion criteria adopted in this study were: patients who had a first-ever ischemic stroke within the 6-0 months leading up to data collection, were undergoing outpatient follow-up, and individuals that agreed to take part. The exclusion criteria adopted were: patients presenting serious psychiatric illness and/or dementia, serious language comprehension or production (aphasia) deficits or that refused to take part.

None of the patients were in use of antidepressive medication and/or submitted to cognitive rehabilitation during the months between hospital discharge and data collection.

**Procedures – Neuropsychological tests.** All study participants were submitted to a neuropsychological assessment protocol, taking a mean time of two hours, carried out on the day they were seen at the neurological outpatient unit. The protocol included collection of sociodemographic data, application of neuropsychological test, functional ability questionnaire and depression inventory. Sociodemographic and clinical data were collected using an identification form filled out by the patient or a family member, as well as by drawing information from the medical chart.

Given that the sample size was only 45 patients and the cross-sectional study was not part of a larger study, where cognitive screening was the only feasible approach for detection of cognitive and follow-up (13), and in view of the possibility of employing a control group, it was decided to carry out a comprehensive neuropsychological evaluation covering all cognitive domains to detect more subtle cognitive impairments. This was the rationale for not using screening tests such as the Mini-Mental State Examination^14^ or the Montreal Cognitive Assessment (MoCA).^15-17^ Lam et al. (2013) suggested care when interpreting an individual's cognitive status based on the MoCA alone, which is a screening test designed to provide an initial indication of the need for more in-depth neuropsychological assessment.^18^

**Instruments.** The following cognitive domains were assessed in the neuropsychological evaluation: reasoning, episodic declarative memory (verbal and visuospatial), attention, neglect, verbal functioning, visuospatial organization and executive functions. The tests used in each domain have been described elsewhere^19^ and are listed in Appendix 1.

For the assessment of functional ability, the Pfeffer Functional Activities Questionnaire (PFAQ) was used, allowing identification of functional compromise based on the presence of difficulty in performing one or more instrumental activities of daily living (food shopping, money management, transport use, preparing meals, using the telephone, taking medications, carry out light and heavy domestic tasks) or a sum of items totaling five or greater (≥5).^20^ The reason for using the PFAQ was its ability to determine the level of independence of the patient for everyday activities in performing tasks that demand recruitment of cognitive resources. Therefore, data collection was not performed using the modified Rankin Scale, which measures functional ability in terms of motor skills as opposed to a cognitive aspects.^21^

The presence of depression symptoms was determined using Beck's Depression Inventory (BDI).^22,23^ For stroke survivors, the scale adopts a cut-off of 10 points to indicate possible depression.^24^

**Statistical analysis.** The general characteristics of the casuistic were expressed as mean and standard deviation for the quantitative variables: age, years of schooling, months since stroke, stroke severity (NIHSS on admission), the functional activities questionnaire and Beck's Depression Inventory. Absolute and relative frequency were used to express the qualitative variables.

The outcome variable "cognitive performance" was obtained by calculating z scores. Raw results from each neuropsychological instrument were transformed into z scores, based on the means and standard deviation obtained with 54 individuals from the community who comprised the control group. Control subjects, matched for age and schooling, were submitted to the same neuropsychological assessment administered to the patients. Cognitive impairment was defined as a z score of ≤ 1.50 standard deviations, indicative of neuropsychological deficit.^25^ Eight cognitive domains were then established: Reasoning, Verbal Memory, Visual Memory, Attention, Hemineglect, Verbal Functions, Visuospatial Organization and Executive Functions. An index was established for each cognitive domain, based on mean of z scores for the neuropsychological tests related to the domain. Overall cognitive performance was obtained based on a mean value, calculated as the sum of the means of each domain divided by the number of domains

After stratification of participants according to cognitive performance (impairment or no impairment), their respective sociodemographic and clinical data, as well as patient performance on the neuropsychological evaluation, depression inventory and functional ability questionnaire, were compared using Student's *t*-test for quantitative variables and the Chi-Square test for qualitative variables. Subsequently, logistic regression was performed with "cognitive performance" as the dependent variable, and sociodemographic and clinical characteristics, functional ability and mood as independent variables. Variables with p<0.10were included in the logistic regression model. Strength of association was measured using odds ratio (OR) and 95% confidence intervals (95% CI).

The level of significance adopted was 5% and SPSS version 16.0 software^26^ was employed for statistical analyses.

## RESULTS

According to the Joinville Stroke Databank Registry, there were 672 cases of stroke at the service of origin between July 2009 and October 2010. However, specific data could only be accessed for the period spanning from October 2009 to October 2010. Over this period, a total of 625 cases of stroke were registered (353 cases among men – 56.48%). In general, the age group most predominantly affected was 60-69 years, accounting for 166 cases (26.56%). The majority of patients had studied up the fourth grade of Primary Education: 234 cases (37.44%), followed by 214 cases with up to three years of formal education (34.24%). Regarding stroke severity, as measured by the NIHSS on admission, most cases (275; 44%) attained a score of between 0 and 4. In terms of etiological classification (TOAST) at hospital discharge, this was classified as undetermined in 179 (28.64%), cardioembolic in 152 cases (24.32%), and lacunar in 144 cases (23.04%). With regard to stroke subtype, there were 265 cases (42.4%) of partial anterior circulation syndromes (PACS).

Mean age in the general group of patients participating in this study was 60 years and 73% of participants were male. Mean schooling was 5 years. Among the study subjects, 71.1% were married and 57.8% retired. The most frequent vascular risk factors were arterial hypertension (68.9%), smoking (48.9%), followed by diabetes mellitus type II (20%) and alcohol use (20%). With the exception of alcohol use, the other vascular risks identified in this study were the same as those observed in a population-based study conducted in the city in 2005 and 2006.^27^ None of the patients presented atrial fibrillation, a vascular risk factor strongly associated with cognitive impairment,^28^ probably because this study involved a younger population. Mean time since stroke was 7.5 months. Stroke severity, as measured by the NIHSS, averaged 7.17 points. In relation to physiopathological diagnosis (TOAST), the majority of cases were atherothrombotic stroke (29 cases, 64.5%). Most patients had supratentorial lesions (40 cases, 88.9%). Lesions to the right hemisphere were detected in 24 cases (53.4%) and to the left hemisphere in 17 cases (37.8%). Based on Bamford's criteria, most strokes were classified as PACS (22 cases, 48.9%) followed by 10 cases of POCS (22.2%).

Comparison of the characteristics of the two groups (cognitive impairment and no cognitive impairment) revealed that the cognitively impaired group had higher mean age (p=0.002), greater stroke severity (p=0.005) and compromised functional ability (p=0.004). Schooling level was greater in the group without cognitive impairment (p=0.042).

No statistically significant difference was found between the two patient groups for time since stroke or depression symptoms. Mean time since stroke event was 7.56 months for the general group of patients. Mean score attained on the BDI, also in the general group, was 7.13 points.

Sociodemographic variables, including socioeconomic level,^29^ marital status and retirement had no statistically significant association with cognitive impairment. The clinical variables, including having undergone thrombolysis or otherwise, physiopathological diagnosis (TOAST), site of lesion, affected hemisphere, stroke subtype (Bamford), and the vascular risk factors of Systemic Arterial Hypertension, Diabetes Mellitus, Dyslipidemia, previous Transient Ischemic Attack, migraine sufferer, alcohol use, tobacco use, Chronic Cardiac Insufficiency, Angina, Acute Myocardial Infarction, Intermittent Claudication, Peripheral Occlusive Arterial Disease, having undergone endarterectomy or otherwise, and use of birth control pills, also showed no statistically significant association with cognitive impairment.

However, having female gender (p=0.002), lower schooling (p=0.042), greater severity stroke (p=0.005) and impaired functional ability (p=0.045) were the characteristics most associated with cognitive impairment.

For the logistic regression, categorical variables with p<0.10were included in the model. The variables found to be associated with cognitive performance were sex (OR=28.995), age (OR=6.815), stroke severity (OR=0.064) and functional ability (OR=6.473).

Subjects in the general group showed worse performance on neuropsychological tests compared to the 54 healthy controls from the community, except on the Trail Making B Test, for the time parameter (p=0.495).

With regard to the neuropsychological profile, the general group of subjects exhibited greater impairment in verbal memory, attention, verbal functions, hemineglect and visuospatial organization. The most affected domains in the group with cognitive impairment were: attention, visuospatial organization, verbal functions and verbal memory.

## DISCUSSION

The aim of this study was to assess cognitive outcome and identify risk factors for impaired cognitive performance in a group of first-ever stroke survivors followed at the specialized out-patient unit of the Unified Health System (SUS); and to describe the neuropsychological profile of the group presenting with cognitive impairment. The rate of cognitive impairment found six months post-stroke was 37.8%, consistent with figures reported in the literature. Nys et al. (2005) found that 30.6% of the patients studied had cognitive impairment in at least one domain, six months post-stroke.^2^ Serrano, Domingo, Rodríguez-Garcia, Castro, and Del Ser (2007) reported 26.8% cognitive impairment 12 months after stroke.^30^

In a review article, Cumming, Marshall and Lazar (2013)^31^ reported the study of Tatemichi et al. (1994) which detected cognitive impairment in all cognitive domains, where those most affected were attention, memory, language and orientation.^32^ The authors also cited a comprehensive European study which identified greatest cognitive impairment in the domains of attention, visuospatial abilities and verbal fluency.^33^ This review noted that stroke currently has greatest impact on attention and executive functions. The disparate results found today are due to the fact that the earlier studies cited above investigated cognitive status of hospitalized patients and healthy controls, whereas current studies tend to be population-based. Consequently, earlier studies may have overestimated the frequency of cognitive deficits in patients.^31^ The present study retains the same design as earlier investigations (patients treated at specific out-patient unit compared to control subjects from the community) and shows that the most common impairments are to verbal memory, attention, verbal functions (including verbal fluency), hemineglect and visuospatial organization. Thus, the pattern of cognitive impairments observed here was the same as those identified in the above-cited studies.^32,33^

Although men predominated in the present casuistic, women had the most severe cognitive impairment. In fact, women had a 21-fold greater risk for cognitive impairment. Other studies^2,32^ have also observed this association. Elderly women may be more vulnerable to cognitive impairment owing to vascular risk factors. The study by Millán-Callenti et al. (2009) in a population aged over 60 years found that females had a greater likelihood of having cognitive impairment, associated with the presence of dementia, heart failure, anemia, stroke and auditory problems.^34^

In the present study, more advanced age was associated with a 4.3 times higher risk for cognitive impairment. Nys et al. (2007) found advanced age to be associated with poorer cognitive recovery at 6-10 months post-stroke.^35^ Raz, Rodrigue, Kennedy and Acker (2007) noted that vascular issues, such as pathological changes in the integrity of white matter and the presence of vascular risk factors were associated with age-related cognitive decline.^36^

Greater stroke severity was also associated with cognitive impairment. Possibly, serious strokes may have more sequelae that hamper recovery. Srikanth et al. (2003) showed an association between cognitive impairment and mild to moderate stroke severity.^37^ Severity of strokes was determined using the NIHSS scale which includes a cognitive component. The study by Cumming et al. (2013) observed that this scale proved a predictor of dementia at 18 months post-stroke.^31^ The study of Kauranen et al. showed that the NIHSS score at hospital discharge was strongly correlated with cognitive impairment. In the same study, 41% of patients with very low NIHSS scores ≤ 4, and all those with scores ≥4, presented cognitive impairment.^38^

Our study showed that the presence of neuropsychological impairment affects the functional abilities of the patient, particularly instrumental activities of daily living. The ARCOS study (2004) found 7% survivors 20 years after stroke, 1/5 of whom required assistance in daily living activities, particularly women and older individuals.^39^ In a review, Feigin et al. (2008) affirmed that functional recovery of stroke survivors on basic daily living activities is possible in the presence of neuropsychological deficits, but this does not mean they have no impairments in instrumental activities of daily living, which demand a higher level of physical functioning.^7^ Cognitive impairment can therefore represent an obstacle to independent life post-stroke.^40^

Given that stroke generally results in psychological stress and limitation in activities involving multiple domains of functioning, the study of stroke should include assessment of multiple functions, thereby allowing the impact of stroke to be accurately quantified and understood as a whole.^6^ Thus, investigating cognitive functioning, presence of depression symptoms and functional ability after stroke is vital for the implementation of interventions in neuropsychological rehabilitation aimed at social reintegration of the patient. However, the specific characteristics of individuals who may suffer greater cognitive impairment following stroke have only recently been elucidated and remain largely inconclusive.^41^ The results of studies on neuropsychological outcome and those predicting cognitive recovery in strokes represent useful information for assessing the cost-benefit of neuropsychological intervention programs.^2,6^ Determining predictors of long-term outcome of stroke survivors can allow the identification of which patients can benefit from certain treatment and rehabilitation strategies, and enable the provision of more consistent information to patients and their family members on the patient's potential for recovery and chances of long-term survival.^7^

Finally, the patients in the present study who had cognitive decline were predominantly of female gender, older, with greater stroke severity and lower functional ability. Based on these findings, the difficulties faced by these patients upon returning to community living can be inferred. This knowledge could allow family members and caregivers to be better informed concerning the cognitive sequelae and the resultant restrictions in independence they impose, given that the cognitive impairments are not always evident. Such information can therefore support the neuropsychological interventions needed, helping patients to achieve greater independence, reintegrate socially and enhance their quality of life.

The main limitations of the present study include the small sample size, the non-inclusion of the Barthel Index for measuring functionality and possible overestimation of cognitive deficits due to the study design. The implications of the study are the inclusion of neuropsychology into out-patient inter-disciplinary teams, whose role will be investigating fundamental aspects in the recovery of stroke and contributing toward improved quality of life of patients.

Future studies involving long-term follow-up of stroke survivors should be conducted, investigating whether the occurrence of a single stroke signals dementia onset, which in turn is associated with a loss of functional ability.

## Figures and Tables

**Table 1:** Comparison of patients exhibiting cognitive impairment and no cognitive impairment by sociodemographic, clinical, functional and mood characteristics of sample (categorical and quantitative variables).

Variables		Total patients n = 45 (100%)	Cognitive impairment (CI) n = 17 (37.8%)	No cognitive impairment (NCI) n = 28 (62.2%)	p
Sex	Male	33 (73.3)	8 (47.1)	25 (89.3)	0.002
	Female	12 (26.7)	9 (52.9)	3 (10.7)	
Age, years	Mean (SD)	59.96 (13.17)	67.47 (12.54)	55.39 (11.51)	0.002
	≥ 60 years	24 (53.3)	13 (76.5)	11 (39.3)	0.050
	40-59 years	14 (31.1)	3 (17.6)	11 (39.3)	
	18-45 years	7 (15.6)	1 (5.9)	6 (21.4)	
Educational Level, years	Mean (SD)	5.02 (3.56)	3.65 (2.74)	5.86 (3.79)	0.042
	> 8 years	7 (15.6)	0 (0.0)	7 (25.0)	0.059
	5-8 years	11 (24.4)	6 (35.3)	5 (17.9)	
	≤ 4 years	27 (60.0)	11 (64.7)	16 (57.1)	
Stroke Severity (NIHSS on admission)	Mean (SD)	6.02 (4.28)	8.24 (5.15)	4.68 (3.03)	0.005
	0-6	32 (71.1)	9 (52.9)	23 (82.1)	0.079
	7-15	12 (26.7)	7 (41.2)	5 (17.9)	
	16-42	1 (2.2)	1 (5.9)	0 (0.0)	
Functional Ability (Performance on Activities of Daily Living Questionnaire)	Mean (SD)	5.69 (7.47)	9.65 (8.91)	3.29 (5.29)	0.004
	With impairment	18 (40.0)	10 (58.8)	8 (28.6)	0.045
	Without impairment	27 (60.0)	7 (41.2)	20 (71.4)	

**Table 2:** Final multiple logistic regression model best explaining association between cognitive impairment and variables.

Variables		Total patients n = 45 (100%)	Cognitive impairment (CI) n = 17 (20.0%)	No cognitive impairment (NCI) n = 28 (80.0%)	OR (95% CI)	p
Sex	Male	33 (73.3)	8 (47.1)	25 (89.3)	28.995 (2.115 - 397.523)	0.012
	Female	12 (26.7)	9 (52.9)	3 (10.7)		
Age	≥ 60 years	24 (53.3)	13 (76.5)	11 (39.3)	6.815 (1.235 - 37.603)	0.028
	40-59 years	14 (31.1)	3 (17.6)	11 (39.3)		
	≤ 45 years	7 (15.6)	1 (5.9)	6 (21.4)		
Educational level	> 8 years	7 (15.6)	0 (0.0)	7 (25.0)	0.447 (0.105 - 2.160)	0.337
	5-8 years	11 (24.4)	6 (35.3)	5 (17.9)		
	≤ 4 years	27 (60.0)	11 (64.7)	16 (57.1)		
Stroke severity (NIHSS on admission)	16-42	1 (2.2)	1 (5.9)	0 (0.0)	0.064 (0.007 - 0.627)	0.018
	7-15	12 (26.7)	7 (41.2)	5 (17.9)		
	0-6	32 (71.1)	9 (52.9)	23 (82.1)		
Functional ability	With functional impairment	18 (40.0)	10 (58.8)	8 (28.6)	6.473 (1.016 - 41.230)	0.048
	Without functional impairment	27 (60.0)	7 (41.2)	20 (71.4)		

**Table 3:** Neuropsychological characteristics of sample and control group (quantitative variables).

Variables		Total patients n = 45 (100%)	Controls (C) n= 54	p
Similarities^1^	Mean (SD)	10.33 (6.68)	14.35 (7.13)	0.012
RAVLT^2^ mean (SD)	A1	3.42 (1.63)	4.93 (1.82)	<0.0001
	A5	7.62 (2.88)	10.11 (2.47)	<0.0001
	Total	29.38 (9.71)	40.28(10.61)	<0.0001
	A6	5.00 (3.10)	7.63 (3.19)	<0.0001
	A7	4.67 (3.49)	7.65 (3.02)	<0.0001
	Recognition	10.96 (3.11)	12.56 (1.97)	0.006
ROCFT^3^ mean (SD)	Copying	17.88 (10.26)	27.84 (5.46)	<0.0001
	Immediate memory	8.11 (7.12)	12.07 (6.05)	0.002
	Delayed memory	8.69 (7.62)	12.75 (5.33)	0.002
Mental control^4^ mean (SD)		11.73 (4.36)	16.26 (4.01)	<0.0001
Numbers^5^ mean (SD)	Direct	6.22 (2.25)	7.15 (1.84)	0.012
	Indirect	2.89 (1.67)	4.06 (1.71)	0.004
	Total	9.11 (3.46)	11.20 (3.13)	0.003
Trail Making Test^6^ mean (SD)	Trail Making A Time	111.29 (85.78)	61.72 (24.85)	<0.0001
	Trail Making A Connections	19.11 (8.52)	23.96 (0.19)	<0.0001
	Trail Making B Time	180.93 (155.27)	164.69 (72.53)	0.446
	Trail Making B Connections	8.87 (9.19)	17.46 (7.44)	<0.0001
Cancellation Task^7^ mean (SD)	Time	269.73 (162.39)	225.69 (65.59)	0.002
	Omissions E	2.82 (5.71)	0.76 (1.01)	0.001
	Omissions D	2.33 (4.12)	0.91 (1.19)	0.003
Phonemic Verbal Fluency^8^ FAR mean (SD)	F	6.18 (3.84)	10.39 (3.38)	<0.0001
	A	5.40 (3.70)	8.98 (3.79)	<0.0001
	S	5.20 (3.51)	9.07 (3.75)	<0.0001
	R	5.20 (3.24)	8.76 (3.74)	<0.0001
	Total (F+A+R)	16.80 (9.88)	28.43 (9.92)	<0.0001
Semantic Verbal Fluency^9^	Animals Category Mean (SD)	9.31 (3.98)	12.19 (2.61)	<0.0001
Token Test^10^ mean (SD)		8.76 (4.04)	11.87 (1.91)	<0.0001
Boston Naming Test^11^ mean (SD)		32.27 (13.91)	42.43 (9.54)	<0.001
Clock Drawing Test^12^ mean (SD)		6.62 (3.07)	8.37 (1.98)	<0.002
Modified Wisconsin Card Sorting test^13^ mean (sd)	Categories	1.60 (0.91)	2.19 (0.70)	0.002
	Errors	8.11 (4.51)	6.69 (4.17)	0.263
	Perseverative Errors	4.20 (3.69)	3.41 (2.62)	0.311
	%	50.99 (33.31)	49.68 (31.16)	0.928
Stroop Test^14^ mean (SD)	I			
	Time	36.09 (19.50)	27.00 (16.24)	0.045
	Errors	1.16 (2.01)	0.35 (0.73)	0.027
	II			
	Time	39.78 (23.54)	26.26 (8.55)	0.001
	Errors	1.53 (2.24)	0.39 (0.79)	0.002
	III			
	Time	53.09 (30.68)	38.19 (10.81)	0.001
	Errors	3.89 (4.48)	2.46 (2.49)	<0.0001

1 Reasoning - verbal abstraction: Similarities subtest (WAIS-III subtest).

2 Rey Auditory Verbal Learning Test.

3 Rey-Osterrieth Complex Figure Test.

4 Mental Control (WMS-III subtest).

5 Numbers (WAISIII subtest).

6 Trail Making Test - form A (time).

7 Cancellation task with unstructured letters (time), cancellation task with unstructured letters (number of omissions to left),cancellation task with unstructured letters (number of omissions to right).

8 Phonemic Verbal Fluency Test (COWA).

9 Semantic Verbal Fluency Test (animals category).

10 Token Test.

11 Boston Naming Test.

12 Clock Drawing Test.

13 Modified Wisconsin Card Sorting Test.

14 Stroop Test.

**Table 4:** Neuropsychological characteristics of sample (categorical variables).

Variables		Total Patients* n = 45 (100%)	Cognitive impairment (CI) n = 17 (37.8%)	No cognitive impairment (NCI) n = 28 (62.2%)
Reasoning^1^	With changes	5 (11.1)	4 (23.5)	1 (3.6)
	Without changes	40 (88.9)	13 (76.5)	27 (96.4)
Verbal memory^2^	With changes	20 (44.4)	13 (76.5)	7 (25.0)
	Without changes	25 (55.6)	4 (23.5)	21 (75.0)
Visual memory^3^	With changes	15 (33.3)	12 (70.6)	3 (10.7)
	Without changes	30 (66.7)	5 (29.4)	25 (89.3)
Attention^4^	With changes	20 (44.4)	15 (88.2)	5 (17.9)
	Without changes	25 (55.6)	2 (11.8)	23 (82.1)
Hemineglect^5^	With changes	17 (37.8)	10 (58.8)	7 (25.0)
	Without changes	28 (62.2)	7 (41.2)	21 (75.0)
Verbal functions^6^	With changes	20 (44.4)	14 (82.4)	6 (21.4)
	Without changes	25 (55.6)	3 (17.6)	22 (78.6)
Visuospatial organization^7^	With changes	17 (37.8)	15 (88.2)	2 (7.1)
	Without changes	28 (62.2)	2 (11.8)	26 (92.9)
Executive Functions^8^	With changes	7 (15.6)	7 (41.2)	0 (0.0)
	Without changes	38 (84.4)	10 (58.8)	28 (100.0)

1 Reasoning - verbal abstraction: Similarities subtest (WAIS-III subtest).

2 Verbal Memory: delayed recall Rey Auditory Verbal Learning Test (A7 - RAVLT).

3 Visual Memory: delayed recall of Rey-Osterrieth Complex Figure Test.

4 Attention: Mental Control (WMS-III subtest), Numbers (WAIS-III subtest), Trail Making Test - form A (time).

5 Hemineglect - spatial attention for determining the presence of hemineglect: cancellation task with unstructured letters (time), cancellation task with unstructured letters (number of omissions to left), cancellation task with unstructured letters (number of omissions to right).

6 Verbal Functions: Phonemic Verbal Fluency Test (COWA), Semantic Verbal Fluency Test (animals category), Token Test and Boston Naming Test.

7 Visuospatial Organization: Clock Drawing Test, .Rey-Osterrieth Complex Figure Test (copying).

8 Executive Functions: Modified Wisconsin Card Sorting Test (number of categories), Stroop test (time III/time I), Phonemic Verbal Fluency Test (COWA), Trail making test (number of connections B - number of connections A).

* General Cognitive Performance: mean of z scores for the cognitive domains: Reasoning, Verbal Memory, Visual Memory, Attention, Hemineglect, Verbal Functions, Visuospatial Organization and Executive Functions.

## APPENDIX 1

**Table:** List of neuropsychological tests and other instruments used in the study.

SIMILARITIES Similarities subtest (Weschler Adult Intelligence Scale - WAIS-III)^42^.	RAVLT Rey Auditory Verbal Learning Test^43^.
ROCFT Rey-Osterrieth Complex Figure Test^44^.	MENTAL CONTROL Mental Control subtest (Weschler Memory Scale - WMS-III)^45^.
NUMBERS Numbers subtest (Weschler Adult Intelligence Scale - WAIS-III)^2^.	TRAIL MAKING TEST Trail Making Test^46,47^.
CANCELLATION TASK Cancellation task, unstructured letters^48^.	PHONEMIC VERBAL FLUENCY COWA Test - Controlled Word Association Test^46,49^.
SEMANTIC VERBAL FLUENCY Semantic Verbal Fluency Test (animals category) Category Fluency Task^46,50^.	TOKEN TEST Token Test^46^.
BOSTON NAMING TEST Boston Naming Test^51,52^.	CLOCK DRAWING TEST Clock Drawing Test^53,54^.
MODIFIED WISCONSIN CARD SORTING TEST Modified Wisconsin Card Sorting Test^46,54^.	STROOP TEST Stroop Test^46^.
PFAQ Pfeffer Functional Activities Questionnaire^20^.	BDI Beck Depression Inventory^22-24^.
